# Mobile health behaviour change support system as independent treatment tool for obesity: a randomized controlled trial

**DOI:** 10.1038/s41366-023-01426-x

**Published:** 2023-12-07

**Authors:** Jaakko O. Markkanen, Noora Oikarinen, Markku J. Savolainen, Heta Merikallio, Ville Nyman, Ville Salminen, Teppo Virkkula, Pasi Karppinen, Harri Oinas-Kukkonen, Janne Hukkanen

**Affiliations:** 1 Research Unit of Biomedicine and Internal Medicine, University of Oulu, Oulu, Finland; 2 Medical Research Center Oulu, Oulu University Hospital and University of Oulu, Oulu, Finland; 3 Department of Medicine, Oulu University Hospital, Oulu, Finland; 4 Oulu Advanced Research on Service and Information Systems, University of Oulu, Oulu, Finland; 5 Biocenter Oulu, Oulu, Finland

**Keywords:** Randomized controlled trials, Weight management

## Abstract

**Background/Objectives:**

Digital health interventions are increasingly utilized as an adjunct to face-to-face counselling in the treatment of obesity. However, previous studies have shown inconsistent efficacy when digital interventions are used as stand-alone treatment. The purpose of this study was to investigate whether a mobile health behaviour change support system (mHBCSS) is effective in weight reduction and weight loss maintenance without additional counselling. Furthermore, changes in cardiometabolic risk factors were investigated.

**Methods:**

In this randomized controlled trial, a mHBCSS intervention was conducted for 200 volunteers with obesity (BMI 30–40 kg/m² and age 18–65 years). The study participants were randomly assigned into two groups: immediate access to mHBCSS intervention or wait-list control with access to mHBCSS after 6 months. Anthropometric and metabolic traits were also measured. The primary outcome was weight loss from the baseline to the 6-month visit.

**Results:**

Among 200 participants (88.5% women), mean BMI (SD) was 34.3 kg/m² (2.8) and age 46.5 years (9.5). The retention rate was 98.5% and 89.0% at the 6- and 12-month visits, respectively. At the 6-month visit, those with immediate access to mHBCSS had significantly greater weight loss (−2.5%, 95% CI −3.4 to −1.6, *p* < 0.001) compared with the wait-list control group (0.2%, 95% CI –0.4 to 0.9, *p* = 0.466; between groups *p* < 0.001). Weight loss was maintained until the 12-month time point in the mHBCSS group (−2.1%, 95% CI −3.3 to −0.9, *p* = 0.001). The usage of mHBCSS had no significant effect on metabolic traits.

**Conclusion:**

The mHBCSS as a stand-alone treatment of obesity results in weight reduction and weight loss maintenance with remarkable adherence rate. Further studies are needed to establish how to best implement the scalable and resource-efficient mHBCSS into the standard care of obesity to achieve optimal weight loss results.

## Introduction

Obesity impairs metabolic health and increases morbidity and mortality. High body mass index (BMI) is associated with the risk of coronary heart disease (CHD), hypertension, and type 2 diabetes (T2DM) [1, 2]. Furthermore, beginning from overweight (BMI 25.0–30.0 kg/m²), the higher the BMI, the higher the associated all-cause mortality [3]. However, even a moderate 5% weight loss decreases cardiometabolic risk factors, such as the concentrations of glucose, insulin, and triglycerides [4].

The current treatment of obesity is often unable to provide long-term weight loss as weight regain is common [5]. This is partly due to certain physiological adaptations and environmental factors favouring weight regain. Therefore, permanent health behaviour change is essential for long-term success in maintenance of weight loss [6]. Long-term changes have been achieved by utilizing methods of cognitive behavioural therapy (CBT) and acceptance and commitment therapy (ACT) [7, 8]. Traditionally, obesity treatment guidelines have recommended long-term comprehensive lifestyle interventions provided by face-to-face counselling [9]. However, the healthcare systems’ resources are often insufficient to offer sufficiently intense face-to-face lifestyle counselling for the growing needs of the population. Thus, there is a need for cost-effective and widely available interventions that can lower the burden on the healthcare systems. Digital tools may provide an opportunity to develop new approaches to treat obesity and prevent comorbidities.

Digital treatment tools, also known as health behaviour change support systems [10], may facilitate individuals to make lifestyle changes leading to successful weight loss, although there are also some conflicting results [11] and recurring limitations in the studies [12]. The best weight loss results have been achieved when electronic health (eHealth) interventions have been used in addition to standard care, while stand-alone eHealth interventions have demonstrated more moderate results [12]. The most common behavioural change techniques used in the weight loss eHealth interventions and based on the behavioural change theories, such as CBT and social cognitive theory, are self-monitoring, goal setting, planning, and feedback [13]. Weight loss eHealth interventions with these kinds of evidence-based features are more effective compared with standard eHealth programs offering mainly passive information for the user [12]. However, the ideal combination of different behaviour change techniques to facilitate weight loss and adherence to intervention are yet to established [13]. Typical limitations in previous mobile weight loss intervention studies have included small sample sizes, duration of 3 months or less, and the absence of no-intervention control groups [14, 15]. A short-term approach to weight loss interventions and lack of follow-up periods are problematic as weight regain is common. Furthermore, most of the studies have investigated eHealth intervention as an adjunct to other types of intervention (e.g., standard care), leading to uncertainty of the effectiveness of specific intervention components [12]. Therefore, randomized controlled trials including longer intervention duration and larger sample sizes are urgently needed to determine if eHealth interventions can be effective as a stand-alone treatment for obesity [15, 16].

We have previously developed a digital health behaviour change support system (HBCSS) [10] to be used via web browsers and aimed at long-term weight loss in patients with overweight and obesity by using persuasive systems design (PSD) [17] and aspects of CBT and ACT. Our previous studies demonstrated that in addition to long-term weight loss, the use of HBCSS improved cardiovascular risk factors among its users as compared with controls [18, 19]. To facilitate widespread adoption of HBCSS for treatment of obesity, we have now developed a mobile version of HBCSS (mHBCSS) to be used with mobile devices. In this randomized controlled trial, we investigated the effectiveness of mHBCSS in the treatment of obesity. We hypothesized that participants randomized to use mHBCSS would lose more weight by 6 months than wait-list controls and the weight loss would be maintained until 12 months. Furthermore, it was hypothesized that users of mHBCSS would have greater improvements in cardiovascular risk factors.

## Materials and methods

### Study design

This study was designed as a randomized, open, wait-list controlled two-arm trial. During the trial’s execution, the principles of Good Clinical Practice and appropriate data protection protocols were followed. The participants received both oral and written information about the trial, and written informed consent was obtained. The study design of the trial was approved by the Ethics Committee of the Northern Ostrobothnia Hospital District (approval number 138/2020) and the Finnish Medicines Agency as mHBCSS is an investigational medical device. The trial was registered at ClinicalTrials.gov (Identifier: NCT04558801).

### Participants

The trial participants were recruited by an open invitation sent to the employees of the Oulu University Hospital and the University of Oulu. Also, the invitation was open to any other willing person, including the employees’ friends and family, and the employees of the private companies located on the campus. The inclusion criteria for participants were BMI 30–40 kg/m², age between 18–65 years, access to a mobile phone or tablet, and no use of other weight management programs or software during participation in this study. The exclusion criteria were uncontrolled hypothyroidism, oral corticosteroid therapy, pregnancy and breastfeeding, cardiovascular disease restricting physical activity, lack of Finnish language skills, planned or previously performed bariatric surgery, or use of anti-obesity drugs. The enrolment was performed by study nurses.

### Randomization

**Fig. 1:**
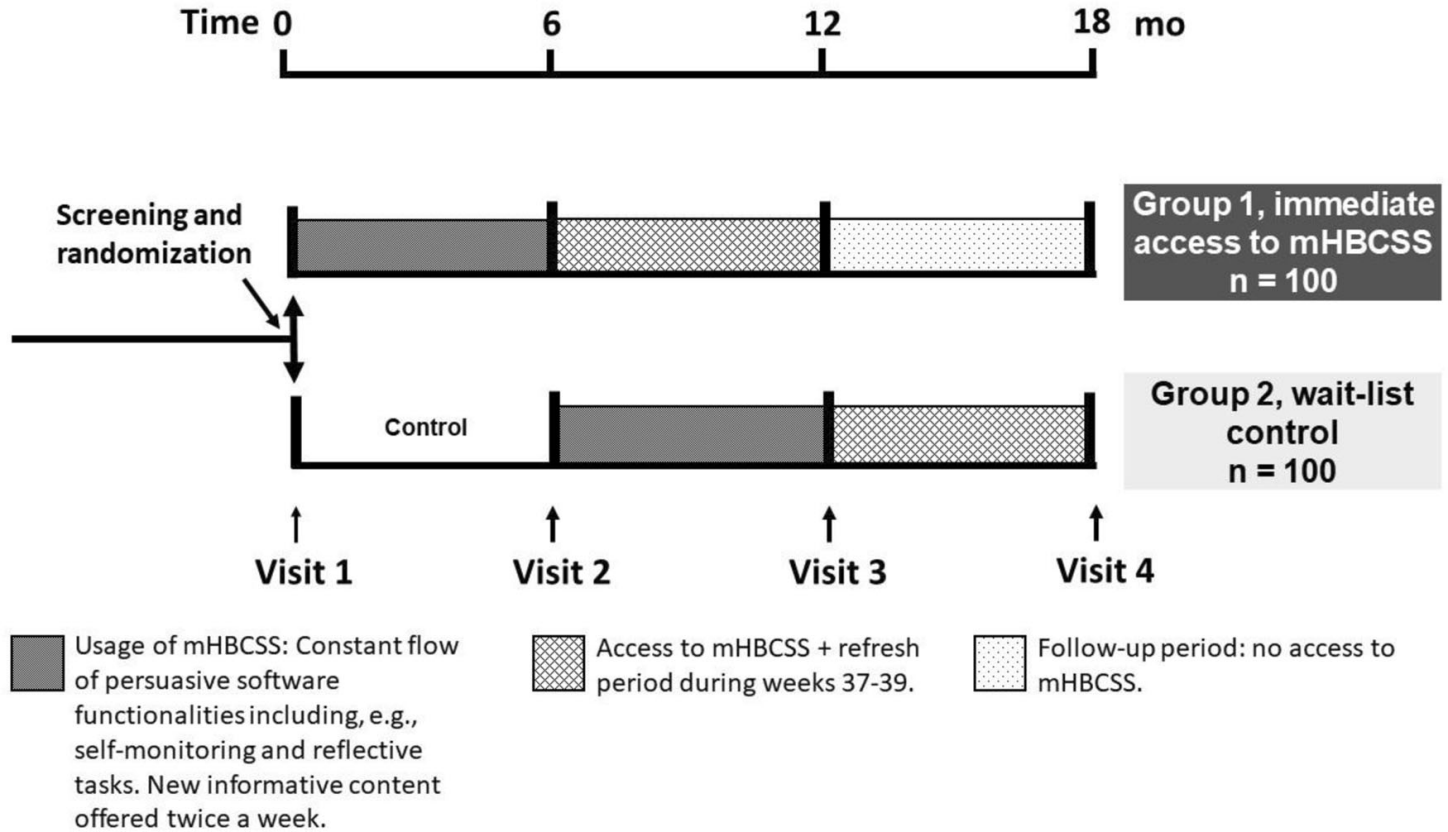
Study design of the clinical trial. Altogether 200 volunteers with obesity (BMI 30 to 40 kg/m^2^, age 18–65 years) were enroled in the study. They were randomized into two groups at the first visit. At each study visit, weight, height, waist circumference and blood pressure were measured, and blood samples were collected to observe metabolic changes.

A randomization list with random permuted blocks of four or eight was prepared by an independent researcher using an online random number generator. All participants were randomly assigned evenly to two groups that received mHBCSS from the beginning of the trial or after a 6-month wait-list time. The study design is illustrated in Fig. 1.

### Interventions

Participants were randomly assigned to two groups by study nurses: immediate access to mHBCSS (Group 1) and wait-list control (Group 2). Group 1 started the 6-month intervention immediately, whereupon mHBCSS supplied articles (informative content) and persuasive software functionalities, e.g., reflective tasks and self-monitoring, for the users twice a week. After this, the users had access to mHBCSS during the following six months and as part of it, a three-week refresh period with some repeated content. The wait-list control group received mHBCSS six months after baseline as having a no-treatment control group or a longer wait before the intervention was deemed unfeasible due to ethical and motivational reasons, and to ensure reaching the recruitment goal in a timely manner. Both groups visited the research unit for measurements at the baseline, at 6 months and 12 months. For both groups, the final study visit will be performed at the 18-month time point (to be reported in a subsequent paper).

Lifestyle counselling was implemented only with the mHBCSS developed by the research group. No additional face-to-face counselling was offered during the trial or during the research visits. The mHBCSS supported weight loss by improving users’ eating behaviour and by facilitating the implementation of other healthy lifestyle changes. To accomplish these aims, mHBCSS utilized PSD and methods of CBT and ACT. The intervention utilises CBT by focusing on thoughts, beliefs and attitudes that affect health-related actions and thereby weight management. ACT is employed in the intervention by coaching the user to contemplate the health-related habits and thoughts, also the unwanted ones, as they are in the moment and focusing on what change the user can commit to. Instead of focusing on problems, intervention focuses on reflection of individual’s own values and aligning health-related actions with these values. The main features during the 6-month intervention were short health-related articles appearing twice a week with occasional small tasks such as learning to recognize dysfunctional thoughts and risky eating situations and contemplating useful coping strategies, self-monitoring activities with varying frequency, and feedback in the form of reminders, suggestions, and praise. The articles provided information related to healthy lifestyle, such as recognition of and coping with thoughts and emotions related to eating behaviour, physical activity, and self-efficacy beliefs. Each article had its own theme such as metabolic syndrome, emotional eating, outdoor exercising, sleep and diet. The occasional tasks included also open and multiple-choice questions regarding each article’s theme, goal setting and other activities that supported processing and the adoption of a healthy lifestyle. The software also provided self-monitoring related functionalities allowing participants to track their own progress. These functionalities included weight monitoring, food and exercise diaries, and a diary where participants could write down their feelings and motivation during the lifestyle change. After recording their weight in mHBCSS, participants automatically received short feedback about their weight loss progress. Participants received a notification of each new article, and they were reminded automatically by a notification if they had not read the article within two days of the article’s release. After 6 months, continuing until 12 months, users had access to mHBCSS and were able to review previously learned topics. In addition, there was a 3-week refresh period (weeks 37–39) with selected content repeated. The refresh period included a total of six articles that appeared twice a week for the users. The first article included personalized feedback according to the self-reported weight change during the intervention, information about weight loss goals and the articles most read by the other users (social aspect). The next four articles had personalized content in line with the individual’s user profile which was generated according to previous answers to questions concerning individual’s eating and exercise habits and thoughts. The last article encouraged individuals to set a new goal related to weight loss or maintenance and reflect on the intervention period.

#### Anthropometric, clinical and biochemical measurements

Height, weight, waist circumference and blood pressure were measured at baseline screening and at 6- and 12-month visits. Blood samples were also collected. Measurements were performed by a study nurse in the research unit of Oulu University Hospital. Participants were given instructions to fast for at least three hours before the blood draws. To measure intervention’s effects on blood parameters related to the increased risk of the metabolic syndrome and cardiovascular diseases by obesity, lipid and glucose values [20] and liver function tests [21] were analysed. The blood samples were analysed at the clinical laboratory of Oulu University Hospital (NordLab, Oulu, Finland). During the study visits, the participants completed a questionnaire about concurrent medications, alcohol use, and previous medical conditions. To reduce diurnal variation, the follow-up visits for each participant were scheduled at the same time of the day as the screening visit. User activity data were logged automatically by the mHBCSS software. From the data, the number of articles read, and the number of weight recordings of each user were examined further.

### Outcomes

The primary outcome for this trial was body weight loss at the 6-month time point. In addition, body weight reduction at the 12-month time point and change in cardiometabolic risk factors including waist circumference, blood pressure, glucose, triglycerides and low-density and high-density lipoproteins at the 6- and 12-month visits were investigated as a secondary outcomes.

### Sample size calculation

Sample size was calculated based on estimation of a 3.2 kg difference between the groups assuming a standard deviation of change of 6.7 for the primary outcome of change in weight. According to this estimation, the sample size required was 69 participants per group. By assuming a dropout rate of 30%, a total of 100 participants per group was estimated to provide 80% power and 5% type 1 error. The a priori assumptions are based on our previous publication on the trial with the web-based HBCSS [19].

### Statistical analyses

The weight and cardiometabolic risk factors of the two groups were compared at three time points: at baseline, 6 months, and 12 months. Variables were compared between groups using the Chi-Square Test or Independent Samples *T* Test as appropriate for the type of data. Within-group changes from baseline to 6 months and to 12 months were analysed using Paired Samples *T* tests. User activity between weight change categories was compared using the Kruskal-Wallis Test. Main analyses were conducted following the intention-to-treat principle (all dropouts included) using baseline values as a substitute for missing data. All statistical analyses were performed using SPSS Statistics (IBM, Armonk, NY, USA). Statistical significance was determined at a two-sided *P* value of <0.05. Normal distribution was evaluated using skewness and kurtosis.

## Results

### Study population

Out of 200 eligible volunteers enroled in the study, only three withdrew from the trial before the 6-month visit. One had started a special diet and decided to discontinue the study. One decided to opt for bariatric surgery, and one could not be contacted. Thus, the 6-month follow-up rate was 98.5%. Between the 6- and 12-month visits, 19 participants withdrew from the study: seven from the immediate access to mHBCSS group and 12 from the wait-list control group. One participant had moved away, one had developed long Covid, and new medication with effects on weight had been prescribed for one participant. In addition, four had not used the mHBCSS and others could not be reached or the reason for withdrawal was unknown. Therefore, the retention rate was 89% (*n* = 178) after 12 months.

### Baseline characteristics

The baseline characteristics of the participants in the two groups are presented in Table 1. The mean (SD) age of the study population was 46.5 (9.5) years, and 88.5% of the study population were women. The mean BMI was 34.3 (2.8) kg/m². There were no significant intergroup differences.

**Table 1:** Baseline characteristics of the participants.

Variables	Group 1, immediate access to mHBCSS	Group 2, wait-list control	*P* value	Total
	(*n* = 100)	(*n* = 100)		(*n* = 200)
Age, year (SD)	46.9 ± 9.9	46.2 ± 9.1	0.599^a^	46.5 ± 9.5
Sex, n (%)				
Males	12 (12)	11 (11)	1.000^b^	23 (12)
Females	88 (88)	89 (89)		177 (89)
Marital status, n (%)				
Married/cohabiting	77 (77)	81 (81)	0.301^b^	158 (79)
Unmarried/divorced/widowed	23 (23)	19 (19)		42 (21)
Educational status, n (%)				
High school graduate	68 (68)	73 (73)	0.535^b^	141 (71)
rCollege or university graduate	72 (72)	73 (73)	1.000^b^	145 (73)
Body weight (kg) (SD)	95.7 ± 12.7	95.2 ± 10.1	0.729^a^	95.4 ± 11.5
BMI (kg/m²) (SD)	34.4 ± 2.8	34.2 ± 2.7	0.616^a^	34.3 ± 2.8
Waist circumference (cm) (SD)	104.3 ± 9.6	104.1 ± 8.2	0.885^a^	104.2 ± 8.9
Systolic blood pressure (mmHg) (SD)	134.7 ± 15.5	132.5 ± 14.3	0.300^a^	133.6 ± 14.9
Diastolic blood pressure (mmHg) (SD)	86.7 ± 8.9	85.5 ± 8.7	0.332^a^	86.1 ± 8.8
Glucose (mmol/l) (SD)	5.6 ± 1.0	5.5 ± 1.5	0.790^a^	5.6 ± 1.2
HbA1c (mmol/l)	36.8 ± 7.5	37.1 ± 10.4	0.780^a^	37.0 ± 9.1
HDL cholesterol (mmol/l) (SD)	1.4 ± 0.3	1.4 ± 0.3	0.923^a^	1.4 ± 0.3
LDL cholesterol (mmol/l) (SD)	3.2 ± 0.8	3.3 ± 0.8	0.475^a^	3.3 ± 0.8
Total cholesterol (mmol/l)	5.1 ± 0.9	5.2 ± 0.9	0.684^a^	5.1 ± 0.9
Triglycerides (mmol/l) (SD)	1.7 ± 0.9	1.7 ± 1.0	0.819^a^	1.7 ± 0.9
AST (U/l)	22.9 ± 16.8	22.3 ± 14.6	0.778^a^	22.6 ± 15.7
ALT (U/l)	34.7 ± 30.6	33.3 ± 26.3	0.731^a^	34.0 ± 28.5
Albumin (g/l)	41.4 ± 2.5	41.1 ± 2.6	0.371^a^	41.3 ± 2.5
Medications, n (%)				
Antihypertensives	39 (39)	28 (28)	0.100^a^	67 (34)
Diabetes	10 (10)	6 (6)	0.300^a^	16 (8)
Cholesterol	11 (11)	7 (7)	0.325^a^	18 (9)

Data are presented as mean ± standard deviation (SD) for continuous variables and numbers (%) for categorical variables.

*mHBCSS* mobile health behaviour change support system, *BMI* body mass index, *HbA1c* glycated haemoglobin, *HDL* high-density lipoprotein, *LDL* low density lipoprotein, *AST* aspartate aminotransferase, *ALT* alanine aminotransferase.

^a^Independent samples *t* test.

^b^Pearson Chi-Square Test.

### Changes in weight and cardiovascular risk factors

From baseline to six months, the Group 1 with immediate access to mHBCSS achieved significantly greater weight change (%) compared to the wait-list control Group 2, the observed difference was 2.7% (95% CI −3.8 to −1.6, *p* < 0.001). At the 6-month visit, significant reduction in body weight (%) was observed within Group 1 but not in the wait-list control Group 2 (Table 2). Regarding the BMI, significant difference was observed between the groups at the 6-month visit (−0.9 kg/m² 95% CI −1.3 to −0.6, *p* < 0.001). At the 12-month point, the weight change was maintained in Group 1 (−2.1%, 95% CI −3.3 to –0.9, *p* = 0.001). Observing the weight change in categories including weight gain, weight loss of <5%, 5–9.99% and over 10%, the difference between the Group 1 and the Group 2 was significant at the 6-month time point (*p* < 0.001) (Table 2).

**Table 2:** Changes in weight and cardiovascular risk factors.

	Change after 6 months					Change after 12 months		
Variables	mHBCSS (*n* = 100)	*P* value^a^	Control (*n* = 100)	*P* value^a^	*P* value^b^	mHBCSS (*n* = 100)	*P* value^a^	*P* value^d^
Weight change (%)	**−2.5** ± **4.5**	**< 0.001**	0.2 ± 3.2	0.489	**< 0.001**	**−2.1** ± **6.0**	**0.001**	0.237
Weight change categories								
Weight loss ≥10%	**6%**		**0%**		**< 0.001**^c^	8%		0.225
Weight loss ≥5–9.99%	**19%**		**7%**			15%		
Weight loss 0–4.99%	**49%**		**42%**			44%		
Weight gain	**26%**		**51%**			33%		
BMI (kg/m²)	**−0.9** ± **1.5**	**< 0.001**	0.07 ± 1.1	0.566	**< 0.001**	**−0.7** ± **2.0**	**< 0.001**	0.314
Waist circumference (cm)	**−2.3** ± **3.7**	**< 0.001**	0.01 ± 2.9	0.972	**< 0.001**	−**2.5** ± **5.0**	**< 0.001**	0.478
Systolic blood pressure(mmHg)	**−4.1** ± **10.5**	**< 0.001**	**−2.5** ± **10.8**	**0.021**	0.286	−1.8 ± 11.1	0.112	**0.022**
Diastolic blood pressure (mmHg)	−**8.2** ± **7.3**	**< 0.001**	−**6.9** ± **7.2**	**< 0.001**	0.191	−**6.2** ± **7.5**	**< 0.001**	**0.006**
Glucose (mmol/l)	−0.1 ± 0.9	0.322	0.03 ± 1.4	0.815	0.470	−0.02 ± 0.8	0.838	0.292
HbA1c (mmol/mol)	−0.8 ± 4.8	0.112	−0.3 ± 7.2	0.669	0.597	−0.5 ± 5.6	0.413	0.227
HDL cholesterol (mmol/l)	−0.01 ± 0.2	0.767	−0.01 ± 0.1	0.561	0.919	0.03 ± 0.2	0.141	0.084
LDL cholesterol (mmol/l)	−0.1 ± 0.5	0.177	−0.07 ± 0.5	0.181	0.989	−0.1 ± 0.5	0.087	0.738
Total cholesterol (mmol/l)	0.0 ± 0.6	0.987	0.05 ± 0.5	0.323	0.509	0.02 ± 0.6	0.728	0.717
Triglycerides (mmol/l)	**−0.2** ± **0.6**	**0.003**	**−**0.1 ± 0.8	0.448	0.218	−0.1 ± 0.9	0.573	0.150
AST (U/l)	−0.9 ± 15.0	0.546	0.04 ± 10.9	0.971	0.609	0.3 ± 13.9	0.841	0.242
ALT (U/l)	**−5.7** ± **24.1**	**0.02**	**−**1.1 ± 14.4	0.439	0.103	−4.6 ± 23.9	0.059	0.375
Albumin (g/l)	−0.4 ± 2.3	0.125	**−**0.4 ± 2.0	0.088	1.000	**−0.5** ± **2.4**	**0.029**	0.403

Data are presented as mean ± standard deviation (SD). Statistically significant changes are in bold.

*mHBCSS* mobile health behaviour change support system, *BMI* body mass index, *HbA1c* glycated haemoglobin, *HDL* high-density lipoprotein, *LDL* low-density lipoprotein, *AST* aspartate aminotransferase, *ALT* alanine aminotransferase.

^a^Within group, paired samples *t* test

^b^Between groups, independent samples *t* test

^c^Between groups, Pearson Chi-Square Test

^d^Within group between the values of 12- and 6-months, paired samples t-test. Intention-to-treat analysis was done by using baseline values for dropouts.

At the 6-month time point, Group 1 had significantly greater reduction in waist circumference compared with the wait-list control group (−2.3 cm, 95% CI −3.2 to −1.4, *p* < 0.001). Furthermore, the reduction in waist circumference was preserved in Group 1 at the 12-month time point. Systolic and diastolic blood pressure was reduced in both study groups from baseline to six months without a significant difference between the groups. In the blood parameters, there were no significant differences observed between the groups at the 6-month visit. The changes in weight and cardiovascular risk factors in the wait-list control group at 12-month visit is presented in supplementary Table 1.

### User activity during 6 months’ use of mHBCSS

**Fig. 2:**
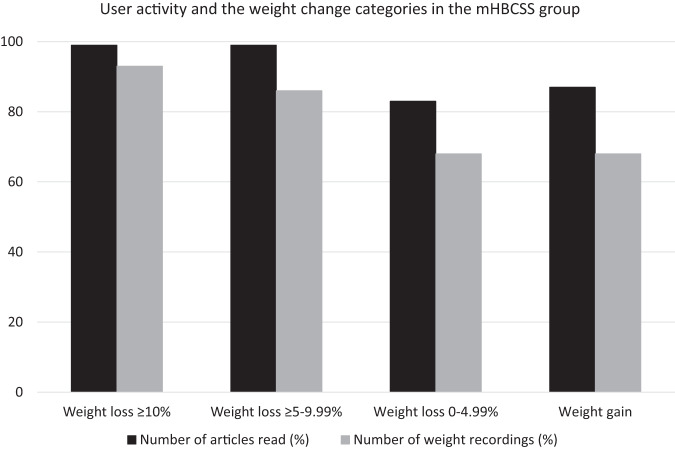
User activity and weight change categories in the immediate access to mHBCSS group. Number of articles read during the 6-month use of mHBCSS by weight change categories (*p* = 0.014) and number of weight recordings during the 6-month use of mHBCSS by weight change categories (*p* = 0.015).

In the group with immediate access to mHBCSS, there were differences in the number of the articles read (*p* = 0.014) and the number of weight recordings (*p* = 0.015) between the four weight loss categories (Fig. 2), with those losing more weight reading more articles and having higher number of weight recordings than those losing less weight.

## Discussion

In this randomized controlled trial, we demonstrate that a mHBCSS is effective as stand-alone treatment for obesity. During the 6-month intervention, the group with immediate access to mHBCSS achieved greater weight reduction than the wait-list control group. Importantly, the weight loss achieved was maintained until 12 months. Moreover, a quarter of the mHBCSS group achieved clinically significant weight loss of 5% or more after six months’ use of mHBCSS.

Without any other intervention component, mHBCSS facilitated weight loss in the group with immediate access to mHBCSS. The weight loss result in the group was similar to the results with other smartphone and stand-alone web-based interventions [22]. Better weight loss results than in our trial have been demonstrated in eHealth intervention studies, where the study settings have had typical limitations for eHealth studies such as low sample size [23–25] or short intervention without follow-up period [24]. For example, six months intervention with MMM app which applies goal setting, self-monitoring (dietary and physical activity) and feedback features built mainly around the calorie counting resulted in weight loss of –4.6 kg (95% CI –6.2 to –3.0) versus –2.3 kg (95% CI –3.1 to –1.5) achieved in this study [23–25]. Weight loss results (reported mean ± SE) were also greater in the 6-month app intervention implemented by diet and step self-monitoring and podcast including behavioural content (–6.8 kg ± 0.8) [23–25]. Both of these studies lacked a no-intervention control group, had no follow-up after the 6-month intervention to explore effects on weight maintenance and had small sample sizes in the app groups (*n* = 43 and *n* = 42, respectively). A more intensive CBT intervention with 19 group meetings delivered in-person during the 6-month intervention demonstrated a greater weight loss result (mean −8.1 kg ± SD 6.8) [26]. This study had no follow-up after intervention and had smaller number of participants (*n* = 95) than our trial.

There is a lack of quality research comparing stand-alone eHealth (application based) interventions to in-person interventions. A non-significant trend on the superiority of the face-to-face counselling over the eHealth interventions has been reported [27, 28]. However, and perhaps most importantly, the most efficient interventions seem to be the ones combining the face-to-face counselling with the eHealth interventions [12, 29, 30]. This was also the result in our previous study with the web-based HBCSS in combination with face-to-face counselling [19]. Thus, for the most optimal weight loss intervention, the future research needs to find the most efficient way to combine these intervention approaches. However, our current study shows that a mHBCSS can be a relevant obesity treatment tool even as a stand-alone therapy, e.g., when the resources limit the availability of face-to-face counselling.

After 12 months, the maintained weight loss result of this study (−2.1%, 95% CI –3.3 to –0.9) achieved by mobile app delivered intervention is comparable with the result with face-to-face counselling observed in one arm of our previous HBCSS study [19] where 6-month face-to-face CBT counselling, including eight clinical nutritionist-delivered sessions and utilizing strategies similar to mHBCSS (building self-efficacy, self-monitoring and feedback), resulted in a weight loss of −1.8% (95% CI −2.9 to −0.6) at the 12-months follow up. The 12-month weight loss result in the group with immediate access to mHBCSS was also comparable to the result of the study arm with the web-based HBCSS as a stand-alone treatment in our previous study (−2.1% vs. −1.4%) [19]. It should be noted that our previous trial involved participants with a lower BMI range (27–35 kg/m^2^) than our current trial (30–40 kg/m^2^).

The mHBCSS aims at long-term changes in body weight by inducing lifestyle changes beneficial for weight loss maintenance [6]. To accomplish these lifestyle changes, mHBCSS utilized principles of CBT, ACT and PSD, all of which have been demonstrated to be effective in the adoption of the behavioural changes needed for sustainable weight loss [7, 31, 32], especially when they are combined in a digital intervention [32]. The behavioural and information systems science approach to weight loss possibly facilitated the sustainable weight loss achieved with mHBCSS in our trial.

A weight loss (between 0 and 10%) achieved by an intervention lasting 6 to 12 months leads to significant improvements in several cardiovascular risk factors, as analysed within-intervention groups in a meta-analysis [33]. In the present study, 74% of the group with immediate access to mHBCSS lost weight during the 6-month mHBCSS intervention. At the 6-month time point, significant improvements were observed in blood pressure and plasma triglyceride levels. However, there was no significant difference in blood pressure and triglycerides when compared between the groups. A significant difference was observed between the groups in the change of waist circumference, which is associated with increased risk of CVDs [34]. In the Look AHEAD trial, a decrease in waist circumference (−9.66 cm ± 0.16), achieved by intensive lifestyle intervention including face-to-face counselling for one year, was associated with lower risk for cardiovascular outcomes compared with participants with increased waist circumference regardless of weight change [35]. Therefore, the sustained decrease in waist circumference achieved by mHBCSS is promising when considering the possible preventive effect of mHBCSS on CVDs.

The importance of patients’ engagement in the intervention for successful weight loss was observed as those in the group with immediate access to mHBCSS who read more articles provided by mHBCSS and recorded their weight more actively had significantly better weight loss results compared with participants with lower user activity. The most effective weight loss application is probably the one that succeeds in engaging patients for the longest time [14]. From this perspective, the mHBCSS had many strengths. The mHBCSS was delivered by an easily accessible mobile application that included engaging features designed by utilizing the principles of PSD [17] and methods of CBT and ACT. In addition to self-monitoring features, the mHBCSS included short and easily performed exercises such as multiple-choice questions that activated participants to reflect on their health behaviour and may have enhanced their engagement with the intervention. Simultaneously, it is remarkable that despite high user activity, 26% of the participants in the mHBCSS group ended up gaining weight (Fig. 2). It is possible that the persons gaining weight with mHBCSS intervention require in-person counselling and more social support to strengthen their self-efficacy towards weight loss process. The reasons behind the interindividual differences in the efficacy of the mHBCSS, and especially weight gain, require further study and are an important topic for our future analyses.

The same strengths of the mHBCSS described above can explain the exceptionally high retention rate in this study. Compared with other behavioural eHealth weight loss intervention studies, most delivered by website or e-mail and including also non-eHealth components (e.g. telephone contacts, face-to-face counselling and written material), this study achieved a remarkably high retention rate at the 6- and 12-month time points compared with the mean retention rate reported in a meta-analysis including 84 trials (98.5% and 89.0% in this study vs. 78% in a meta-analysis) [12]. Interestingly, the retention rate in this study seems to be higher than in other stand-alone digital intervention studies in which one meta-analysis, which excluded hybrid interventions, showed that 7 out of the 11 retrieved studies had attrition rates ≥20% [36]. The retention rate in this mHBCSS study was also higher compared with our previous study on web-based HBCSS and based on the same principles as the mHBCSS (91% vs 81% at the 12-month time-point) [10]. However, it should be noted that the enrolment to our current trial was targeted for people employed at proximity to our research centre which probably enhanced the retention. Smartphones are considered as the most effective platform for eHealth interventions, with higher adherence than in web-based or personal digital assistant interventions [22]. In addition to higher engagement, mobile interventions are easily accessible, with no time-limits for use. Self-management of obesity treatment with the help of mobile interventions could not only help ease the burden on healthcare providers but also be more suitable and pleasant for some patients when compared with traditional intervention modalities, such as face-to-face counselling, which are usually offered only during office hours.

Our study has some limitations. We were not able to have a no-treatment control group for the whole duration of the study as access to mHBCSS was given to the wait-list control group after a 6-month control period for motivational and ethical reasons. We assumed that if the control group had not been given access to mHBCSS, recruitment would have been more difficult and the dropout rate higher. In addition, as in most eHealth studies, blinding was considered unfeasible. Furthermore, the majority of the participants were women employed in the local University and the University Hospital affecting the generalizability of the results.

This randomized controlled trial demonstrates that after a 6-month intervention, a stand-alone mHBCSS that includes evidence-based behavioural change strategies induces a significant weight loss which is maintained up to 12 months. The weight loss was accomplished with minimal resources as the participants did not receive any other type of counselling. Therefore, the stand-alone mHBCSS is a useful, resource-efficient and scalable method to treat obesity when resources to offer face-to-face counselling for obesity are limited or when remote treatment suits better to the patient’s lifestyle. There is a need for studies on the implementation of interventions such as mHBCSS in the standard care of obesity in order to find the most cost-effective ways to achieve maximal long-term weight loss results in the population at large.

### Supplementary information

Supplementary table

Supplementary figure

## Data Availability

The data that support the findings of this study are available on request from the corresponding author. Personal data are not publicly available due to privacy and ethical restrictions.

## References

[1] Holmes MV, Lange LA, Palmer T, Lanktree MB, North KE, Almoguera B (2014). Causal effects of body mass index on cardiometabolic traits and events: a Mendelian randomization analysis. Am J Hum Genet.

[2] Lyall DM, Celis-Morales C, Ward J, Iliodromiti S, Anderson JJ, Gill JMR (2017). Association of body mass index with cardiometabolic disease in the UK biobank: a mendelian randomization study. JAMA Cardiol.

[3] di Angelantonio E, Bhupathiraju SN, Wormser D, Gao P, Kaptoge S, de Gonzalez AB (2016). Body-mass index and all-cause mortality: individual-participant-data meta-analysis of 239 prospective studies in four continents. The Lancet.

[4] Magkos F, Fraterrigo G, Yoshino J, Luecking C, Kirbach K, Kelly SC (2016). Effects of moderate and subsequent progressive weight loss on metabolic function and adipose tissue biology in humans with obesity. Cell Metab.

[5] Blüher M (2019). Obesity: global epidemiology and pathogenesis. Nat Rev Endocrinol.

[6] Greenway FL (2015). Physiological adaptations to weight loss and factors favouring weight regain. Int J Obes (Lond).

[7] Jacob A, Moullec G, Lavoie KL, Laurin C, Cowan T, Tisshaw C (2018). Impact of cognitive-behavioral interventions on weight loss and psychological outcomes: A meta-analysis. Health Psychol.

[8] Lawlor ER, Islam N, Bates S, Griffin SJ, Hill AJ, Hughes CA (2020). Third-wave cognitive behaviour therapies for weight management: A systematic review and network meta-analysis. Obes Rev.

[9] Wadden TA, Tronieri JS, Butryn ML (2020). Lifestyle modification approaches for the treatment of obesity in adults. Am Psychol.

[10] Oinas-Kukkonen H (2013). A foundation for the study of behavior change support systems. Pers Ubiquitous Comput.

[11] Sorgente A, Pietrabissa G, Manzoni GM, Re F, Simpson S, Perona S, et al. Web-based interventions for weight loss or weight loss maintenance in overweight and obese people: a systematic review of systematic reviews. J Med Internet Res. 2017; 19. 10.2196/JMIR.6972. 10.2196/jmir.6972 PMC5504341 28652225

[12] Hutchesson MJ, Rollo ME, Krukowski R, Ells L, Harvey J, Morgan PJ (2015). eHealth interventions for the prevention and treatment of overweight and obesity in adults: a systematic review with meta-analysis. Obes Rev.

[13] Asbjørnsen RA, Smedsrød ML, Nes LS, Wentzel J, Varsi C, Hjelmesæth J, et al. Persuasive system design principles and behavior change techniques to stimulate motivation and adherence in electronic health interventions to support weight loss maintenance: scoping review. J Med Internet Res. 2019; 21. 10.2196/14265. 10.2196/14265 PMC6611151 31228174

[14] Mateo GF, Granado-Font E, Ferré-Grau C, Montaña-Carreras X. Mobile phone apps to promote weight loss and increase physical activity: a systematic review and meta-analysis. J Med Internet Res. 2015;17. 10.2196/JMIR.4836. 10.2196/jmir.4836 PMC4704965 26554314

[15] Semper HM, Povey R, Clark-Carter D (2016). A systematic review of the effectiveness of smartphone applications that encourage dietary self-regulatory strategies for weight loss in overweight and obese adults. Obes Rev.

[16] Wang Y, Xue H, Huang Y, Huang L, Zhang D (2017). A systematic review of application and effectiveness of mhealth interventions for obesity and diabetes treatment and self-management. Adv Nutr.

[17] Oinas-Kukkonen H, Harjumaa M (2009). Persuasive systems design: key issues, process model, and system features. Commun Assoc Inf Syst.

[18] Seo YG, Salonurmi T, Jokelainen T, Karppinen P, Teeriniemi AM, Han J (2020). Lifestyle counselling by persuasive information and communications technology reduces prevalence of metabolic syndrome in a dose-response manner: a randomized clinical trial (PrevMetSyn). Ann Med.

[19] Teeriniemi AM, Salonurmi T, Jokelainen T, Vähänikkilä H, Alahäivälä T, Karppinen P (2018). A randomized clinical trial of the effectiveness of a Web-based health behaviour change support system and group lifestyle counselling on body weight loss in overweight and obese subjects: 2-year outcomes. J Intern Med.

[20] LeRoith D (2007). Dyslipidemia and glucose dysregulation in overweight and obese patients. Clin Cornerstone.

[21] Targher G, Corey KE, Byrne CD NAFLD, and cardiovascular and cardiac diseases: Factors influencing risk, prediction and treatment. Diabetes Metab. 2021; 47. 10.1016/J.DIABET.2020.101215. 10.1016/j.diabet.2020.101215 33296704

[22] Cavero‐Redondo I, Martinez‐Vizcaino V, Fernandez‐Rodriguez R, Saz‐Lara A, Pascual‐Morena C, Álvarez‐Bueno C (2020). Effect of behavioral weight management interventions using lifestyle mhealth self-monitoring on weight loss: a systematic review and meta-analysis. Nutrients.

[23] Carter MC, Burley VJ, Nykjaer C, Cade JE Adherence to a smartphone application for weight loss compared to website and paper diary: pilot randomized controlled trial. J Med Internet Res. 2013;15. 10.2196/JMIR.2283. 10.2196/jmir.2283 PMC3636323 23587561

[24] Martin CK, Miller AC, Thomas DM, Champagne CM, Han H, Church T (2015). Efficacy of SmartLoss, a smartphone-based weight loss intervention: results from a randomized controlled trial. Obesity (Silver Spring).

[25] Turner-McGrievy GM, Wilcox S, Boutté A, Hutto BE, Singletary C, Muth ER (2017). The dietary intervention to enhance tracking with mobile devices (DIET Mobile) study: a 6-month randomized weight loss trial. Obesity (Silver Spring).

[26] Batra P, Das SK, Salinardi T, Robinson L, Saltzman E, Scott T (2013). Eating behaviors as predictors of weight loss in a 6 month weight loss intervention. Obesity (Silver Spring).

[27] Padwal RS, Klarenbach S, Sharma AM, Fradette M, Jelinski SE, Edwards A (2017). The evaluating self-management and educational support in severely obese patients awaiting multidisciplinary bariatric care (EVOLUTION) trial: principal results. BMC Med.

[28] Hurkmans E, Matthys C, Bogaerts A, Scheys L, Devloo K, Seghers J. Face-to-face versus mobile versus blended weight loss program: randomized clinical trial. *JMIR* Mhealth Uhealth. 2018; 6. 10.2196/MHEALTH.7713. 10.2196/mhealth.7713 PMC5785684 29326093

[29] Berry MP, Sala M, Abber SR, Forman EM (2021). Incorporating automated digital interventions into coach-delivered weight loss treatment: a meta-analysis. Health Psychol.

[30] Antoun J, Itani H, Alarab N, Elsehmawy A. The effectiveness of combining nonmobile interventions with the use of smartphone apps with various features for weight loss: systematic review and meta-analysis. JMIR Mhealth Uhealth. 2022; 10. 10.2196/35479. 10.2196/35479 PMC9034427 35394443

[31] Sairanen E, Tolvanen A, Karhunen L, Kolehmainen M, Järvelä-Reijonen E, Lindroos S (2017). Psychological flexibility mediates change in intuitive eating regulation in acceptance and commitment therapy interventions. Public Health Nutr.

[32] Sittig S, McGowan A, Iyengar S. Extensive review of persuasive system design categories and principles: behavioral obesity interventions. J Med Syst. 2020; 44. 10.1007/S10916-020-01591-W. 10.1007/s10916-020-01591-w 32500161

[33] Zomer E, Gurusamy K, Leach R, Trimmer C, Lobstein T, Morris S (2016). Interventions that cause weight loss and the impact on cardiovascular risk factors: a systematic review and meta-analysis. Obes Rev.

[34] Huxley R, Mendis S, Zheleznyakov E, Reddy S, Chan J (2010). Body mass index, waist circumference and waist:hip ratio as predictors of cardiovascular risk-a review of the literature. Eur J Clin Nutr.

[35] Olson KLL, Neiberg RH, Espeland MA, Johnson KC, Knowler WC, Pi-Sunyer X (2020). Waist circumference change during intensive lifestyle intervention and cardiovascular morbidity and mortality in the look AHEAD trial. Obesity (Silver Spring).

[36] Beleigoli AM, Andrade AQ, Cançado AG, Paulo MNL, Diniz MDFH, Ribeiro AL. Web-based digital health interventions for weight loss and lifestyle habit changes in overweight and obese adults: systematic review and meta-analysis. J Med Internet Res. 2019; 21. 10.2196/JMIR.9609. 10.2196/jmir.9609 PMC6330028 30622090

