# Supplementary figures and images for: Mobile health behaviour change support system as independent treatment tool for obesity: a randomized controlled trial
Source: Int J Obes (Lond). 2023 Dec 7;48(3):376–83. doi: 10.1038/s41366-023-01426-x (PMC10896717; doi:10.1038/s41366-023-01426-x)

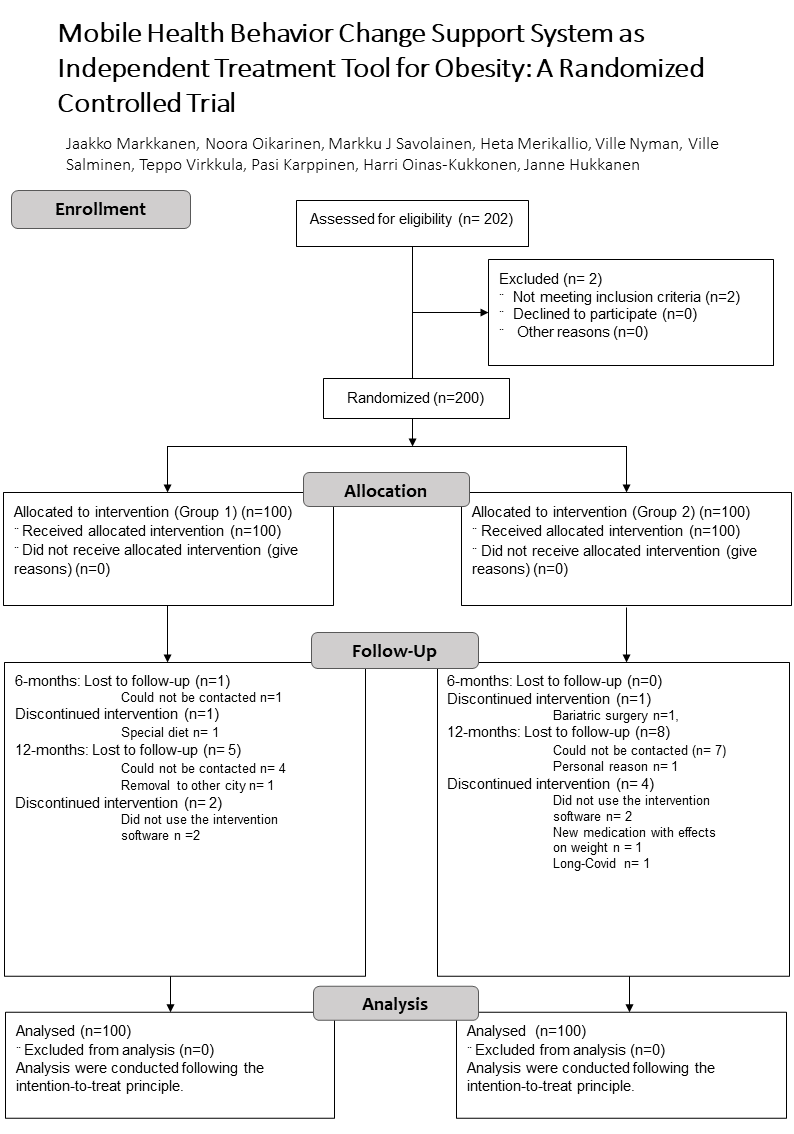

Supplement: Supplementary file 2 — Supplementary figure [file 41366_2023_1426_MOESM2_ESM.tif]
